# Generation and Characterization of a Novel *Prkcd-*Cre Rat Model

**DOI:** 10.1523/JNEUROSCI.0528-24.2024

**Published:** 2024-07-08

**Authors:** Sanne Toivainen, Michele Petrella, Li Xu, Esther Visser, Tamina Weiss, Sofia Vellere, Zane Zeier, Claes Wahlestedt, Estelle Barbier, Esi Domi, Markus Heilig

**Affiliations:** ^1^Department of Clinical and Experimental Medicine, Linkoping University, Linkoping 58225, Sweden; ^2^School of Pharmacy, Center for Neuroscience, Pharmacology Unit, University of Camerino, Camerino 62032, Italy; ^3^Department of Psychiatry and Behavioral Sciences, Center for Therapeutic Innovation, University of Miami Miller School of Medicine, Miami, Florida 33136

**Keywords:** aversion, central amygdala, CRISPR, PKCδ

## Abstract

Activity of central amygdala (CeA) PKCδ expressing neurons has been linked to appetite regulation, anxiety-like behaviors, pain sensitivity, and addiction-related behaviors. Studies of the role that CeA PKCδ+ neurons play in these behaviors have largely been carried out in mice, and genetic tools that would allow selective manipulation of PKCδ+ cells in rats have been lacking. Here, we used a CRISPR/Cas9 strategy to generate a transgenic *Prkcd*-cre knock-in rat and characterized this model using anatomical, electrophysiological, and behavioral approaches in both sexes. In the CeA, Cre was selectively expressed in PKCδ+ cells. Anterograde projections of PKCδ+ neurons to cortical regions, subcortical regions, several hypothalamic nuclei, the amygdala complex, and midbrain dopaminergic regions were largely consistent with published mouse data. In a behavioral screen, we found no differences between Cre^+^ rats and Cre^−^ wild-type littermates. Optogenetic stimulation of CeA PKCδ+ neurons in a palatable food intake assay resulted in an increased latency to first feeding and decreased total food intake, once again replicating published mouse findings. Lastly, using a real-time place preference task, we found that stimulation of PKCδ+ neurons promoted aversion, without affecting locomotor activity. Collectively, these findings establish the novel *Prkcd*-Cre rat line as a valuable tool that complements available mouse lines for investigating the functional role of PKCδ+ neurons.

## Significance Statement

The central nucleus of the amygdala (CeA), involved in processing threat and aversion signals, comprises multiple neuronal subtypes. Expression of protein kinase C isoform δ, PKCδ, marks CeA neurons that respond to aversive stimuli and have also been shown to play a role in alcohol-related behaviors. Genetic tools to investigate the functional role of PKCδ+ neurons in rat models have been lacking. We describe the development and characterization of a novel *Prkcd* knock-in transgenic rat generated using CRISPR strategy. In this model, we confirm known projection targets of CeA PKCδ+ neurons and replicate functional consequences of their activation previously found in mice. This establishes the line as a novel model to study the role of PKCδ+ neurons in rat models.

## Introduction

The central nucleus of amygdala (CeA) is involved in threat, pain, and appetite processing (Kong and Zweifel, 2021). Conceptualization of its function has evolved from a view as a passive relay station between sensory input nuclei of the amygdala and behavioral output centers in the brainstem (LeDoux, 2000), to one of an active node of information processing (Balleine and Killcross, 2006; Ciocchi et al., 2010; H. Li et al., 2013; Robinson et al., 2014; Janak and Tye, 2015; K. Yu et al., 2017). CeA is largely GABAergic and shows high cellular heterogeneity in its subregions (Haubensak et al., 2010; O'Leary et al., 2022). However, establishing the link between activity of specific neuronal subpopulations and CeA function has proven challenging (Wang et al., 2023).

Based on anatomy and gene expression, CeA can be broadly divided into two subdivisions. The centrolateral division (CeL) displays strong intrinsic inhibitory connectivity and acts as a go/no-go relay by modulating the activity of centromedial (CeM) output neurons (Duvarci and Pare, 2014). Most CeL neurons fall into largely nonoverlapping populations expressing protein kinase Cδ (PKCδ, encoded by *Prkcd*) and somatostatin (SOM), respectively (Haubensak et al., 2010; H. Li et al., 2013). PKCδ+ neurons were initially described as OFF cells, based on their inhibitory response to conditioned fear stimuli, while SOM+ neurons were labeled as ON cells, because of their increased firing to the same stimulus (Ciocchi et al., 2010). Development of transgenic *Prkcd*-cre mice has since allowed detailed mechanistic studies on the role played by PKCδ+ CeA neurons. These are activated by unconditioned threat stimuli, and sufficient to generate aversive memories, as their stimulation induces both conditioned and real-time place aversion (RTPA; Cui et al., 2017; K. Yu et al., 2017). Optogenetic inhibition of PKCδ+ CeA neurons has been shown to inhibit fear learning by interfering with transfer of information about the threat stimulus to the lateral amygdala (K. Yu et al., 2017).

CeL PKCδ+ neurons are also involved in behaviors beyond threat processing. For instance, they bidirectionally modulate pain-related behavior in mice. Their inhibition, or inhibition of their projections to the zona incerta, reduces neuropathic hyperalgesia (Singh et al., 2022), while their stimulation increases pain sensitivity (Wilson et al., 2019). PKCδ expression is also enriched in neuronal ensembles activated by itch (Samineni et al., 2021), suggesting that PKCδ+ neurons broadly mediate responses to aversive stimuli. Finally, activation of CeL PKCδ+ neurons leads to a reduction in food intake, while their inhibition promotes feeding (Cai et al., 2014).

A growing literature suggests an important role of CeA PKCδ+ neurons in addiction-related behaviors. We found increased *Prkcd* expression and PKCδ+ neuronal activity in the CeA of rats showing shock-resistant or “compulsive” alcohol self-administration. In this model, selective PKCδ knockdown in the CeA decreased compulsive alcohol self-administration (Domi et al., 2021). A role of CeA PKCδ+ neurons in alcohol-related behaviors is further supported by findings that this population exhibits the highest level of differential gene expression during alcohol withdrawal (Dilly et al., 2022). CeA PKCδ+ neurons may play a role in other addiction-related behaviors, since their activity is required for the protective effects of social interaction to inhibit incubation of craving (Venniro et al., 2018, 2020).

Genetically modified driver mouse lines have been critical for advances in the understanding of CeA function, but additional insights can be obtained using rat models. To that end, we developed and characterized a *Prkcd-*Cre knock-in rat line using a CRISPR/Cas9 strategy (Redman et al., 2016). Using RNAscope in situ hybridization and Cre-dependent adeno-associated viral (AAV) tracing combined with immunohistochemical labeling, we performed an extensive anatomical mapping of PKCδ projections. We also examined a range of spontaneous behaviors to determine whether these were affected by the Cre insertion. Finally, to determine whether our model replicates published mouse findings, we optogenetically stimulated CeA PKCδ+ neurons and examined their role in consumption of palatable food and in real-time place preference.

## Materials and Methods

### Subjects

We used 206 *Prkcd*-hemizygotes (133 males and 73 females) and 174 wild types (88 males and 86 females) for molecular and behavioral experiments. Adult (7–9 weeks) male and female *Prkcd*-Cre rats weighing ∼350 and 250 g, respectively, at the beginning of the experiments were kept group housed (3–4 rats per cage) with *ad libitum* access to tap water and food pellets. Animals were housed in a temperature- and humidity-controlled vivarium on a 12 h light/dark cycle (lights off at 7:00 A.M.). Body weights were monitored weekly, and rats were handled three times before the start of any experimental procedure. All experiments were performed during the dark phase of the light/dark cycle. Experimental procedures were conducted in accordance with the European Union Directive 2010/63/EU, and the protocol was approved by the Ethics Committee for Animal Care and Use at Linköping University (Dnr 16869-2022).

### Rat *Prkcd*::Cre knock-in line

**Figure 1.**
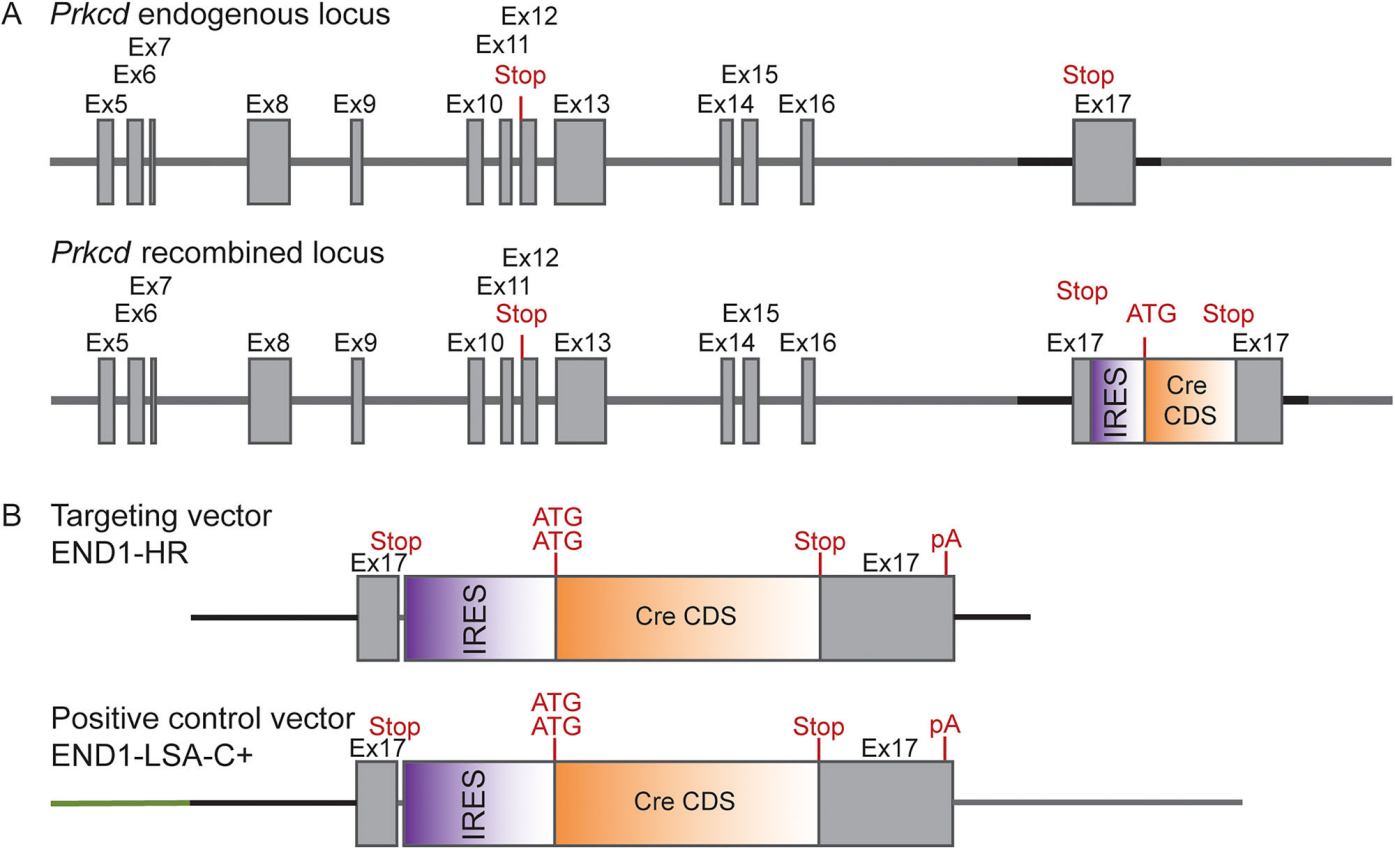
Schematic representation of the selected targeting strategy. ***A***, Gray boxes: *Prkcd* coding sequences. Solid lines: Intronic regions. The locations of the initiation (ATG) and STOP (Stop) codons are indicated. Sequence elements are not depicted to scale. ***B***, Schematic representation of the END1 targeting and control constructs. Orange box: Cre recombinase coding sequence. Purple box: IRES element. Green line: Recombination junction of the vector homology arm and the genomic locus. Sequence elements are not depicted to scale.

PKCδ knock-in Cre rats were generated using CRISPR/Cas9 technology (provider: genOway; Shao et al., 2014). An IRES-Cre cassette was inserted downstream of the STOP codon in exon 17 (Fig. 1*A*). A targeting construct containing the IRES-Cre coding sequence flanked by genomic homology sequences was cloned as shown in Figure 1*B* (top construct). In parallel, a second vector was created, which mimics the DNA conformation at the targeted locus after homologous repair mediated insertion of the targeting cassette, between the short homology arm of the targeting vector and the *Prkcd* locus. The quality of the resulting final END1-HR targeting vector was controlled by sequence analysis.

Both constructs were microinjected into pronuclei of fertilized oocytes and after overnight observation reimplanted into pseudopregnant foster rats. Successful integration of the IRES-Cre cassette was confirmed by PCR screening which tested for the presence of a 0.97 kb PCR product. The integrity of the recombinant locus was confirmed by sequencing the entire target region plus 1 kb downstream and upstream. Mating the founder with Wistar wild-type animal generated the final hemizygous knock-in rats. The presence of the knock-in construct in the offspring was confirmed by PCR and sequencing. Further breeding with Wistar wild-type rats confirmed germline transmission.

### Breeding and genotyping

Male *Prkcd*-Cre hemizygotes were set up in breeding with wild-type Wistar females (Charles River). For each generation, 20 breeding pairs were used. Tail genotyping was performed in-house. Briefly, ear biopsies were collected, and DNA was isolated by addition of 500 µl 50 mM NaOH. Tissue samples were kept on a vibrating heating block (Grant Instruments Europe) at 95°C until the tissue had dissolved, then 60 µl 1 M Tris-HC, pH 7.5, was added to each tube. PCR was performed using REDExtract-N-Amp PCR ReadyMix (Merck) and the following probes: END1-35 5′-TTCCTTCAGTGACAAGAACCTCATCGAT, END1-37 5′-GGCAAATTCACAAACAGTTCACAGAGGA, PanCre-rv 5′-CAGACCAGGCCAGGTATCTCT, PanCre – fw 5′-AGAACCTGATGGACATGTTCAGG. The following program was run on a SimpliAmp thermal cycler (Applied Biosystems, Thermo Fisher Scientific International): Denaturation 94°C 120 s 1×, (Denaturation 94°C 30 s—annealing 65°C 30 s—extension 68°C 300 s)×40, Completion 68°C 480 s, Hold 10°C indefinitely. Lastly, samples were run on an agarose gel at 110 V for 35 min before being analyzed in a gel reader (Bio-Rad).

### RNAscope in situ hybridization and double-labeling immunohistochemistry

Naive animals were perfused with saline followed by 4% PFA. Brains were removed and postfixed in PFA overnight. Then, brains were transferred to 30% sucrose in 1× PBS solution until sinking. Brains were sectioned on a cryostat (16 µm sections for RNAscope, 30 µm sections for immunohistochemistry, AP −2.2 to −2.8 mm from bregma). Sections were kept in cryoprotectant solution (20% glycerol and 30% ethylene glycol in 1× PBS), until further processing.

In situ hybridization was performed using the RNAScope Kit V2 (Advanced Cell Diagnostics). Sections were washed twice in 1× PBS at room temperature and then mounted onto Superfrost Plus Gold Adhesion microscope slides (Epredia Netherlands). Sections were air-dried and then baked for 1 h at 60°C. Subsequently, sections were postfixed in 10% NBF for 15 min at 4°C, followed by 5 min dehydration steps in 50, 70, 100, and 100% ethanol at room temperature. Sections were treated with hydrogen peroxide for 10 min at room temperature. Target retrieval was performed by first submerging the slides for 10 s in boiling distilled water and then for 5 min in boiling target retrieval buffer. Slides were immediately submerged in distilled water and transferred to 100% ethanol for 3 min. Slides were air-dried until drawing a hydrophobic barrier around the sections. The next day, sections were treated with Protease Plus for 30 min at 40°C, followed by washes in distilled water. Then, sections were incubated with the target probe for *Prkcd* mRNA [C2, diluted in Tyramide Signal Amplification (TSA) buffer; Advanced Cell Diagnostics, catalog #573261-C2] for 2 h at 40°C in the HybEZ oven (Advanced Cell Diagnostics). Sections underwent amplification steps (AMP1, AMP2 30 min and AMP3 15 min at 40°C). Then, the HRP-C2 channel was developed, by incubation with HRP-C2 for 15 min at 40°C, followed by fluorophore incubation [TSA Plus Cy3 (TS00202), Akoya Biosciences; 1:2,000 in TSA buffer] for 30 min at 40°C and HRP blocker for 15 min at 40°C. Excess liquid was removed and DAPI applied for 30 s at room temperature, immediately followed by mounting using ProLong Diamond Antifade Mounting Medium (P36961, Invitrogen) and coverslipping. Sections were dried for 30 min and then stored at 4°C until imaging 3 d later.

Double-labeling immunohistochemistry was performed as described previously (Domi et al., 2021). Briefly, floating brain sections were washed in PBS three times for 10 min and then blocked in a solution of 4% bovine serum albumin (BSA) and 0.2% Triton X-100 dissolved in PBS for 1 h at room temperature. For PKCδ labeling, a monoclonal mouse anti-PKCδ (1:500 dilution; 610398, BD Biosciences) was used as the primary antibody. Sections were incubated in primary antibodies for 24 h at 4°C. After being rinsed in PBS three times, the sections were incubated with Alexa Fluor 647 goat anti-mouse secondary antibody (1:200 dilution; A-21236, Invitrogen) for 2 h at room temperature. Sections were again rinsed in PBS three times and mounted on slides and coverslipped with ProLong Diamond Antifade Mountant with DAPI (P36962, Invitrogen). Sections were dried for 30 min and then stored at 4°C until imaging.

### Image acquisition and processing

Sections were imaged using a Zeiss LSM800 confocal microscope with a 20× objective. Two to four images per animal were obtained. For RNAscope, images were analyzed using QuPath version 0.4.2 (Bankhead et al., 2017) to quantify the number of *Prkcd* mRNA spots per DAPI-labeled cell. The number of *Prkcd-*positive spots were quantified using the subcellular detection feature with the following parameters: expected spot size, 3 µm; minimal spot size, 1.4 µm; and maximal spot size, 8.0 µm, including clusters. The quantification of PKCδ and cre-dependent mCherry expression cells was processed with ImageJ software. Two to four images per animal were obtained. Individual cells were identified based on DAPI staining of the nucleus. The percentage of cells expressing PKCδ and cre-dependent mCherry was determined by dividing the number of PKCδ-positive cells by the total number of cre-dependent mCherry-expressing cells and vice versa in the same image.

### Baseline phenotyping

#### Developmental screening

Basic neurological tests were performed in pups between postnatal days (P) 4 and 20, adapted from a battery of tests developed for mutant mice (Crawley, 2007). Animals were assessed for developmental milestones such as pinnae detachment, incisor eruption of upper and lower jaw, fur development, and eye opening. Further, pups were screened for surface righting reflexes by placing them on the back and measuring the time until the they turned over to right themselves. Next, to evaluate negative geotaxis, pups were placed face down on a 45° inclined surface, and the time until they changed orientation to face up was measured. For tests of surface righting and negative geotaxis, a 60 s cutoff was implemented. To evaluate the grasp reflex, pups were held by the scruff, a thin rod was stroked against the paw, and the ability of the pups to grasp the rod was recorded. Visual placing response was measured by holding pups by the trunk and bringing their head toward a colored cloth on a flat surface. The extension of the paws toward the colored surface was noted. Lastly, tactile and auditory startle reflexes were measured by recording jumping or twitching responses to an air puff onto the back of the pup or in response to the sound of a noise. No interaction with the pups occurred before P4 to minimize stress of the dam.

#### Locomotor activity

Locomotor activity was tested using automated locomotion recording chambers (44.5 cm × 44.5 cm × 30.5 cm; Med Associates) equipped with 10 equally spaced infrared (I/R) light beams and inserted into sound-attenuating cubicles. Activity was recorded during a single session. Animals were placed in the center of the chamber and allowed to freely explore for 30 min. Locomotor activity was automatically analyzed in 5 min intervals.

#### Anxiety-like behavior: elevated plus maze

Anxiety-like behavior was assessed using the elevated plus maze (EPM) as previously described (Domi et al., 2018). Briefly, the animal was placed in the center of the maze, facing a closed arm, and allowed 5 min to freely explore the arena. The percentage of time spent in the open arms (% open time) was used as a measure of anxiety-like behavior. Behavior was scored by trained experimenters blind to genotype.

#### Learning and memory: novel object recognition task

Test was performed in a 1 × 1 m arena containing custom-made objects (Barchiesi et al., 2022). Animals were allowed 10 min to habituate to the arena containing two identical copies of either object A or B. Half of the animals were exposed to object A and the other half to object B. The following day, they were tested for novel object recognition by being placed in the arena for 5 min with one of the familiar objects having been replaced with one that is novel. Time spent exploring each object was scored by a trained experimenter blind to genotype and expressed as a recognition index, defined as time spent exploring novel object / (time spent exploring novel + familiar object).

#### Pain sensitivity: hot plate test

The hot plate test was performed using Ugo Basile apparatus; the test consisted of placing the animal on a heated surface with a constant temperature of 52°C and measuring the latency to first lick their forepaws or the latency to try to jump out of the apparatus. Each animal was tested twice. The test was performed by two operators blinded to genotype. The cutoff time was 60 s, if the animal failed to react within this time the test was considered invalid, and the animal was taken out of the apparatus. Between animals, the apparatus was cleaned and reset to the initial temperature.

#### Pain sensitivity: tail flick test

The tail flick test was performed using the D’Amour and Smith method (D'Amour and Smith, 1941; Ugo Basile apparatus). The animals were restrained manually with their tails left extended. The test consisted of focusing a beam of light on three different positions along the tail base. The light would confer radiant heat, and the latency to flitch the tail outside of the apparatus was monitored. A cutoff of 10 s was maintained; if the animal did not show painful reaction within this set time, the trial was considered invalid, and the animal would be removed from the apparatus.

#### Corticosterone levels

Blood samples were collected from the tail vein immediately after the onset of the dark phase of the light cycle. Samples were centrifuged for 15 min at 4°C, 2,000 × *g* per minute, and plasma was extracted. Corticosterone was extracted as described previously (Domi et al., 2021). A corticosterone enzyme immunoassay kit (Arbor Assays, Nordic Biosite) was used to analyze the samples for corticosterone levels. Detection levels for corticosterone were 7.8–1,000 ng/ml.

### Surgery

Animals were anesthetized with isoflurane (∼4% induction, ∼2% maintenance) and injected with buprenorphine (0.03 mg/kg, s.c.). Carprofen (5 mg/kg, s.c.) was injected on the day of surgery and 2 d postsurgery. Rats were mounted into a stereotactic frame, and Cre-dependent viral vectors were targeted at the CeA using the following coordinates: AP: −2.75; ML: ±4.50; DV: −8.45 from bregma. For electrophysiological experiments, AAV_9_-*EF1a*-DIO-hChR2(H134R)-mCherry (Addgene, 20297-AAV9, titer 1 × 10^13^) was used, and for anterograde tracing, we infused AAV_5_-*hSyn*-DIO-mCherry (Addgene. 50459-AAV5, titer 7 × 10^12^). For the in vivo optogenetic experiment AAV_5_-*hSyn*::DIO-EGFP (Addgene, 50457-AAV5, titer 7 × 10^12^, Addgene, control) or AAV_5_-*hSyn*::DIO-ChR2-EYFP (Addgene, 26973-AAV5, titer 2.5 × 10^13^) was injected. Viral vectors were bilaterally infused using a modified cannula at a flow rate of 100 nl/min, with a total volume of 500 nl per hemisphere. After allowing an additional 5 min for diffusion, the cannula was retracted stepwise. For optogenetic experiments an optic fiber [flat tip, 200/250 µm; numerical aperture (NA), 0.66; Doric Lenses] was implanted above the CeA (AP: −2.75; ML: ±4.50; DV: −8.15 from bregma). Three stainless steel screws (Protech International) were inserted into the skull as anchors, and the implanted optic fiber ferrule was fixed using black carbon-mixed dental cement (Ortho-Jet; Lang Dental). Animals recovered from surgery in their home cage and remained there for at least 3 weeks to ensure sufficient viral expression before the start of experiments. In the week prior to behavioral testing, rats were handled and habituated to the fiber patch cord connection three times.

### Electrophysiological recordings

Adult male and female *Prkcd*-cre rats were deeply anesthetized with isoflurane and decapitated 4–8 weeks after AAV injections. Brains were quickly removed and placed into an ice-cold *N*-methyl-d-glucamine (NMDG)-based cutting solution (Ting et al., 2014) containing the following (in mM): 92 NMDG, 20 HEPES, 25 glucose, 30 NaHCO_3_, 1.2 NaH_2_PO_4_, 2.5 KCl, 5 sodium ascorbate, 3 sodium pyruvate, 2 thiourea, 10 MgSO_4_, and 0.5 CaCl_2_ (310 mOsm), pH 7.4. Acute coronal brain slices (250 μm thick) containing the CeA or the PAG were obtained using a vibratome (Leica VT1200 S, Leica Biosystems). After cutting, slices were transferred to a holding chamber filled with a prewarmed (∼34°C) holding solution containing the following (in mM): 92 NaCl, 20 HEPES, 25 glucose, 30 NaHCO_3_, 1.2 NaH_2_PO_4_, 2.5 KCl, 5 sodium ascorbate, 3 sodium pyruvate, 2 thiourea, 1 MgSO_4_, and 2 CaCl_2_ (310 mOsm), pH 7.4. Subsequently, the holding solution was maintained at room temperature, and after >1 h of recovery, a single slice was transferred to the recording chamber and continuously perfused at a flow rate of ∼2.0 ml/min with warmed (∼30–32°C) artificial cerebrospinal fluid (aCSF, in mM): 125 NaCl, 2.5 KCl, 1.25 NaH_2_PO_4_, 1 MgCl_2_, 11 glucose, 26 NaHCO_3_, 2.4 CaCl_2_ (310 mOsm), pH 7.4. All solutions were saturated with 95% O_2_ and 5% CO_2_. Neurons were visualized with infrared differential interference contrast (IR-DIC) optics on a Zeiss Examiner.A1 microscope (Carl Zeiss). Electrophysiological recordings of optogenetically evoked IPSCs (oIPSCs) were carried out using borosilicate glass patch pipettes (2.5–3.0 MΩ; Harvard Apparatus) containing the following (in mM): 140 Cs-methanesulfonate, 5 NaCl, 1 MgCl, 10 HEPES, 0.2 EGTA, 2 Mg-ATP, 0.5 Na-GTP, 5 QX-314 chloride (290 mOsm, pH adjusted to 7.3 using CsOH). Recorded neurons were held at 0 mV, and photocurrents were evoked by 5 ms blue light pulses delivered at a frequency of 0.1 Hz. Light-evoked GABAergic currents were blocked by perfusion of the GABA_A_ receptor antagonist picrotoxin (100 μM). Peak current amplitude was measured using the average of at least 15 subsequent photostimulation sweeps. On current-clamp recordings, pipettes were filled with a solution containing the following (in mM): 135 K-gluconate, 20 KCl, 10 HEPES, 0.1 EGTA, 2 MgCl_2_, 2 Mg-ATP, 0.3 Na-GTP (290 mOsm, pH adjusted to 7.3 using KOH). PKCδ+ and PKCδ− neurons were identified by the presence or absence of mCherry fluorescence and ChR2-evoked currents (evoked by a 1 s blue light stimulation pulse at the end of recordings), respectively. Recordings were performed using a MultiClamp 700B amplifier (Molecular Devices), digitalized with a Digidata 1440A (Molecular Devices; 2 kHz low-pass Bessel filter and acquired 20 kHz), and acquired with pClamp 10.7 software (Molecular Devices). The estimated junction potential was 11 mV for K-Gluc and 8.5 mV for Cs-based intracellular solution and was not compensated during electrophysiological recordings.

### Probing the functional role of CeA PKCδ+ neurons

#### Optogenetic stimulation during palatable food intake

*Ad libitum* fed animals were first habituated to chocolate-flavored cereal (Nesquik Original Cereal, Nestlé) consumption for six sessions (first session, 30 min; subsequent sessions, 15 min) in an open-field–like arena (50 cm × 50 cm × 50 cm) with food presented in a Petri dish. During the last two habituation sessions, animals were connected to the fiber patch cord without stimulation, to habituate the animals to the fiber. The testing phase consisted of two sessions (Pretest, Test) lasting a total of 30 min. Palatable food intake was measured by weighing food pellets and crumbs before the test, 15, and 30 min after the test began. During the pretest, animals were connected to the fiber patch cord, without stimulation. The next day, during the test, animals were exposed to light stimulation (470 nm LED, 10 Hz, 10 ms pulse, at 8–9 mW; Prizmatix) during the first 15 min of the session, and no stimulation during the last 15 min. One animal lost its optic fiber implant after the feeding experiment and was therefore excluded from the remainder of the study.

#### Biased real-time place aversion test

Rats were tested in a two-chamber arena [70 cm (35 cm each chamber) × 30 cm × 50 cm]. Rats first underwent a pretest session in which they could freely explore the full arena for 10 min to measure innate individual preference. During the pretest, animals were connected to the fiber patch cord in the absence of light stimulation. The following day on test, animals were exposed to the same conditions but received optogenetic stimulation (470 nm LED, 10 Hz, 10 ms pulse, at 8–9 mW) upon entering the previously preferred chamber.

#### Open field

The open field test was carried out in a 1 × 1 m arena, and rats were allowed to freely explore the arena for 15 min in presence of optogenetic stimulation (10 Hz, 10 ms pulse, at 8–9 mW). The distance traveled was recorded and automatically analyzed using an EthoVision XT video-tracking system (Noldus).

### Statistical analysis

Data were analyzed with STATISTICA, Stat Soft 13.0 (RRID:SCR_014213), using analysis of variance (ANOVA), with number of animals, sex, factors, and degrees of freedom for the respective analysis indicated in conjunction with its results. Prior to ANOVA, data were examined for assumptions of homogeneity of variance and normality of distribution using Levene's and Shapiro–Wilk test, respectively. Where homogeneity of variance or normality were significantly violated, data were square root transformed. Statistically significant difference was set at *p* < 0.05. Post hoc analyses were conducted when appropriate using the Newman–Keuls test. The data are presented as the mean ± SEM.

## Results

### Insertion of Cre into the *Prkcd* locus results in selective Cre activity in CeA PKCδ+ neurons

**Figure 2.**
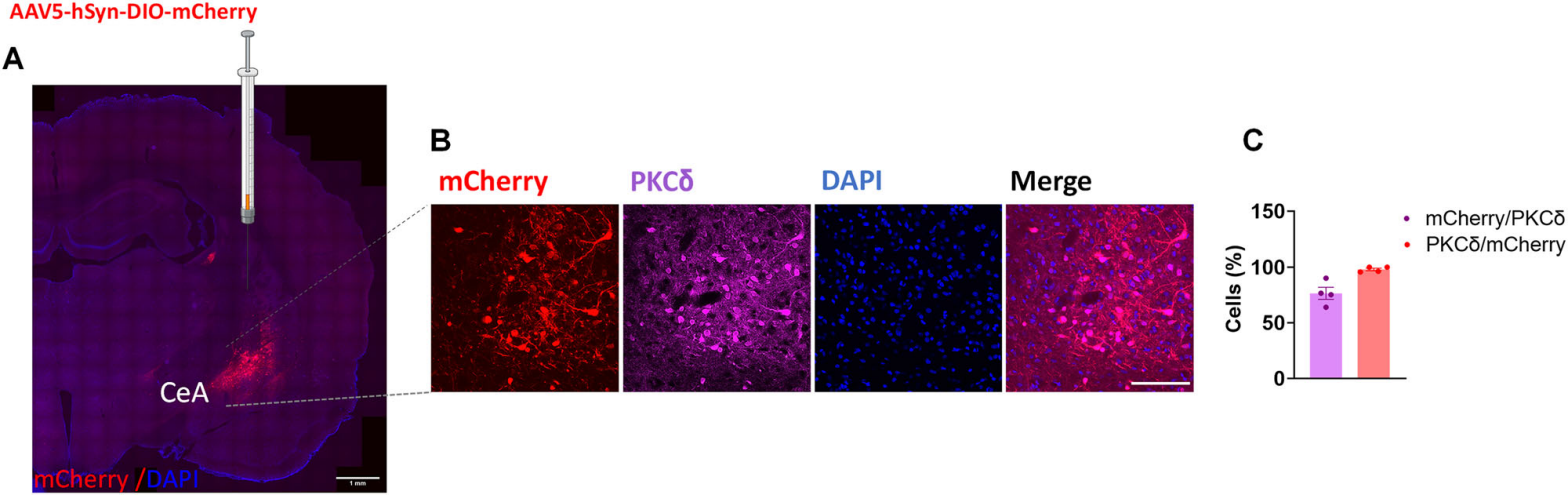
Anatomical validation of CRISPR-mediated knock-in of *Prkcd*. ***A***, Virus injection site. Scale bar, 1 mm. ***B***, Representative images of CeA photomicrographs showing mCherry immunoreactivity (red), PKCδ (magenta), and their colabeling in *Prkcd* Cre knock-in rats (*n* = 4). ***C***, Mean ± SEM immunoreactive PKCδ, mCherry + neurons expressed in % number of cells. Scale bar, 50 μm.

To evaluate the selectivity and function of Cre expression resulting from our targeting strategy, male *Prkcd-*Cre rats were injected with a virus encoding Cre-dependent mCherry into the CeA. After allowing sufficient time for vector expression and recombination, we assessed PKCδ and mCherry coexpression using immunohistochemistry (Fig. 2). The majority of PKCδ+ neurons also expressed mCherry (76.58 ± 5.3%; *n* = 4), and virtually all mCherry-expressing neurons were positive for PKCδ immunodetection (98 ± 1.2%; *n* = 4).

### Expression of PKCδ following Cre insertion into the *Prkcd* locus

**Figure 3.**
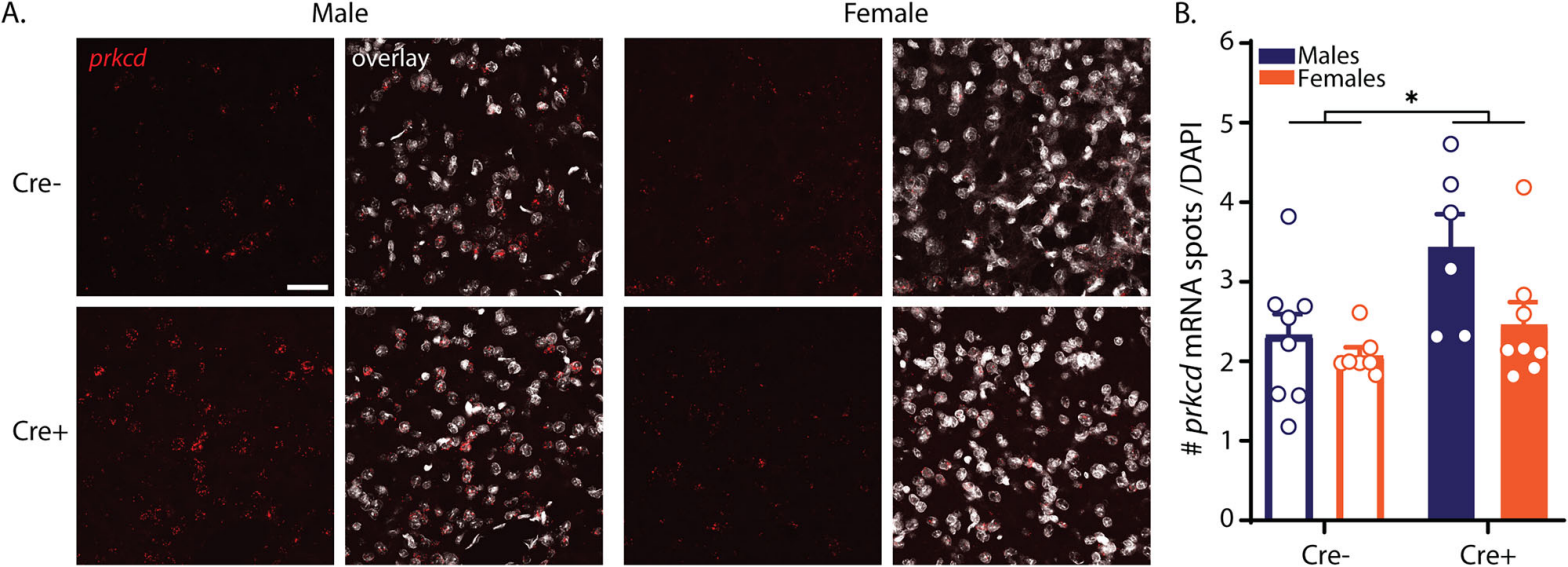
Cre insertion increased *Prkcd* expression in the centrolateral amygdala of male rats. ***A***, Representative images of *Prkcd* mRNA (in red) with DAPI (in cyan) in the centrolateral amygdala. Scale bar, 50 μm. ***B***, Quantification of the number of *Prkcd* mRNA spots, normalized to DAPI for wild-type Cre^−^ (open bars; *n *= 8 males, *n *= 7 females) and transgenic Cre^+^ animals (filled bars; *n *= 6 males, *n *= 8 females). *p = 0.014.

To assess whether insertion of the Cre-cassette affected endogenous *Prkcd* expression, we performed RNAScope in situ hybridization, focusing on the centrolateral amygdala (CeL; Fig. 3). We observed an increased number of *Prkcd* mRNA spots in transgenic animals (Cre^+^) compared with wild-type littermates (Cre^−^; main effect of genotype *F*_(1,25) _= 6.940; *p* = 0.014), with a trend for increased expression in males compared with females (main effect of sex *F*_(1,25) _= 4.160; *p* = 0.052), and no interaction between the two factors (*F*_(1,25) _= 1.678; *p* = 0.207). Šídák's multiple-comparisons post hoc test showed a significant increase in the number of *Prkcd* mRNA spots in transgenic male rats compared with male wild-type Cre^−^ littermates (*p* = 0.023), while no genotype difference was found in females.

### Anterograde tracing of CeA PKCδ+ neurons

**Figure 4.**
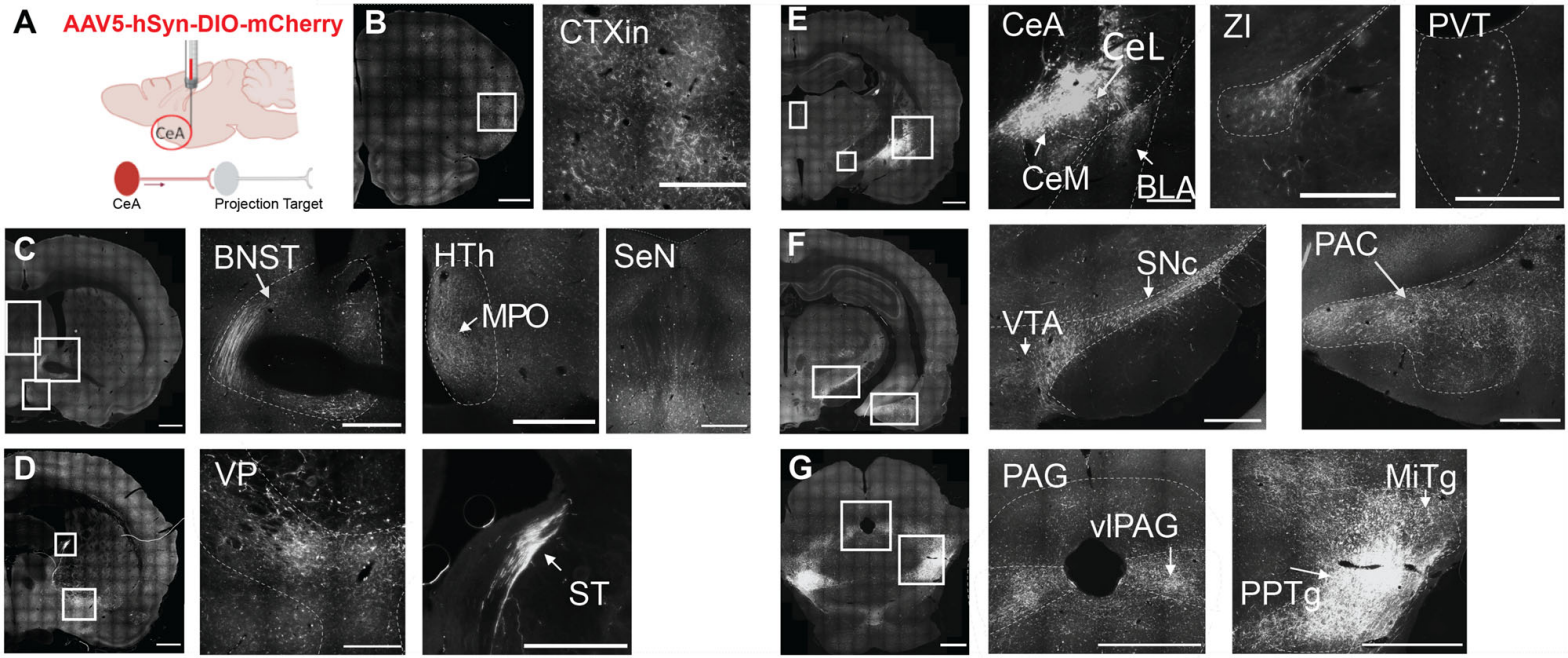
Projection targets of PKCδ+ CeA neurons. ***A***, Schematic of the approach, injection site and viral tracing in *Prkcd-*Cre knock-in rats (*n* = 4); bregma levels and anatomical landmarks are provided for the output projections. ***B–G***, Epifluorescence microscopy images of the areas containing PKCδ+ axons labeled by mCherry. Fluorescent signal is shown in gray scale from caudal to rostral (top row to bottom row). The first image of each panel shows the half/full-coronal brain sections (scale bar, 1 mm) followed by high magnification images (scale bar 500 µm). BLA, basolateral amygdala; BNST, bed nucleus of the stria terminalis; CeA, central nucleus of the amygdala; CeL, lateral division; CeM, medial division; CTXin, insular cortex; HTh, hypothalamus; MiTg, microcellular tegmental nucleus; MPO, medial preoptic area; PAC, periamygdaloid cortex; PAG, periaqueductal gray; PPTg, pedunculopontine tegmentum; PVT, paraventricular thalamus; SeN, septal nuclei; SNc, substantia nigra pars compacta; ST, stria terminalis; vlPAG, ventrolateral periaqueductal gray; VP, ventral pallidum; VTA, ventral tegmental area; ZI, zona incerta.

CeA PKCδ+ neurons are known to exert a strong local control over CeA microcircuits and function (Duvarci and Pare, 2014). However, they also send long-range projections to several regions throughout the brain, and some of those have been ascribed a specific functional role (K. Yu et al., 2017; Singh et al., 2022). To evaluate the projection pattern of rat CeA PKCδ+ neurons, we bilaterally injected male Cre^+^ rats with a Cre-dependent virus to selectively express mCherry in PKCδ+ neurons and in their terminal (Fig. 4*A*). mCherry-positive terminals were widely distributed throughout the brain. Projections were *i.a.* found in cortical regions, such as the insular cortex (CTXin; Fig. 4*B*). CeA PKCδ+ neurons also innervated several subcortical areas, such as the bed nucleus of the stria terminalis (BNST), various nuclei of the hypothalamus, especially the medial preoptic area (MPO), septal nuclei (Fig. 4*C*), the ventral pallidum (VP), the paraventricular thalamus (PVT), the zona incerta (ZI), the amygdala complex (i.e., the CeM and the basolateral amygdala, BLA; Fig. 4*D*), as well as midbrain dopaminergic regions, including the ventral tegmental area (VTA) and the substantia nigra pars compacta (SNc; Fig. 4*E*). Fibers were also found to target multiple nuclei of the periamygdaloid cortex (PAC). Lastly, we further observed strong projections toward the ventrolateral column of the PAG (vlPAG) as well as the mesopontine tegmentum (MPTg), especially the subnuclei pedunculopontine tegmentum (PPTg) and microcellular tegmentum (MiTg; Fig. 4*F*). These findings are highly consistent with the projection patterns previously observed for mouse CeA PKCδ+ neurons (K. Yu et al., 2017).

### Insertion of Cre into the *Prkcd* locus does not alter spontaneous behaviors or corticosterone levels

#### Developmental screening

To assess whether insertion of the Cre cassette would disrupt normal physiological and neurological development, a screen was performed in an independent cohort of Cre^+^ and Cre^−^ littermates from age P4 until P17. Pups were evaluated for a series of behaviors (Table 1), and the span of postnatal days in which the behavior was observed in any pups was recorded. Furthermore, the day in which >50 and 75% of pups had acquired the behaviors was recorded. A failed attempt was noted for an animal when >75% of the other pups in the same litter had reached a milestone. The proportion of failed attempts were as follows: male Cre^−^, 0.25 (5/20); male Cre^+^, 0.25 (5/20); female Cre^−^, 0.2 (4/20); and female Cre^+^, 0.15 (3/20).

**Table 1.** Developmental screening of Cre^+^ and Cre^−^ littermates

Milestone	Span (days)	>50% acquired (days)	>75% acquired (days)
Pinnae detachment	P1–4	N/A	N/A
Incisor eruption	P7–11	P9	P10
Fur development	P8–11	P9	P10
Eye opening	P13–17	P15	P15
Surface righting	P4–8	P4	P6
Negative geotaxis	P4–8	P5	P6
Tactile startle	P8–12	P9	P10
Bar holding	P8–12	P9	P10
Visual placing	P13–16	P15	P15
Auditory startle	P14–16	P15	P15

Developmental screening evaluated between P4 and P17 for male Cre^−^ (*n* = 20), male Cre^+^ (*n* = 20), female Cre^−^ (*n* = 20) and female Cre^+^ (*n* = 20) animals.

**Figure 5.**
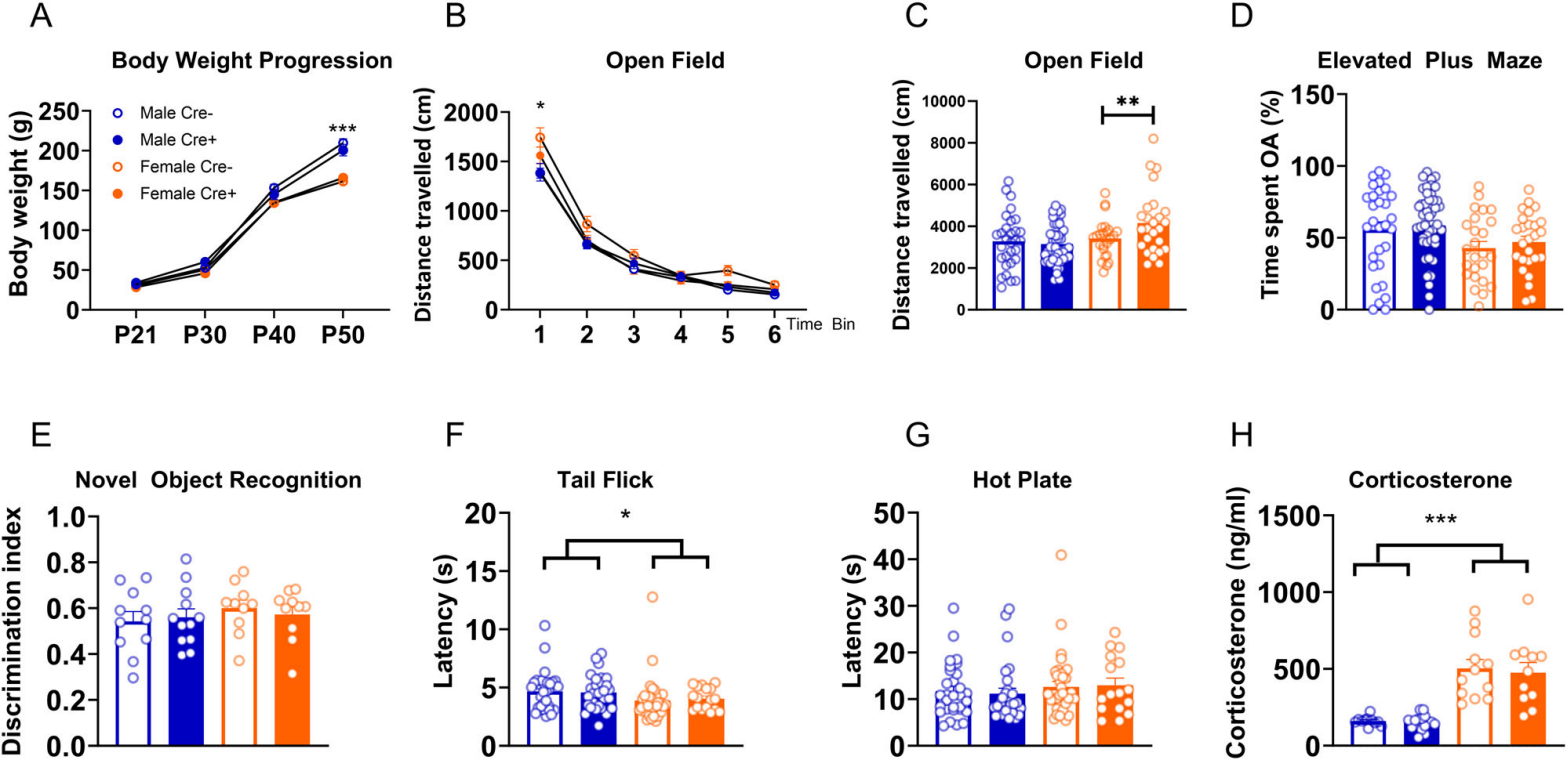
The knock-in of Cre into the *Prkcd* gene does not alter basal behaviors and corticosterone levels. ***A***, Body weight progression measured across P21–P50 for male Cre^−^ (*n* = 20), male Cre^+^ (*n* = 20), female Cre^−^ (*n* = 20), and female Cre^+^ (*n* = 20) animals. ***B***, Locomotor activity measured as distance traveled in an open field-like arena and total distance traveled (***C***). Male Cre^−^ *n* = 30; male Cre^+ ^= 47; female Cre^−^ *n* = 25; female Cre^+^ *n* = 26. ***D***, Anxiety-like behavior, measured as proportion of time spent in the open arm of the EPM. Male Cre^−^ *n* = 30; male Cre^+ ^= 47; female Cre^−^ *n* = 25; female Cre^+^ *n* = 26. ***E***, Short-term memory function, as measured by discrimination index in a novel object recognition test. Male Cre^−^ *n* = 11; male Cre^+ ^= 12; female Cre^−^ *n* = 10; female Cre^+^ *n* = 10. ***F***, ***G***, Pain sensitivity, as measured by latency to remove tail in the tail flick (***F***, male Cre^−^ *n* = 30; male Cre^+ ^= 28; female Cre^−^ *n* = 34; female Cre^+^ *n* = 17) and hot plate (***G***, male Cre^−^ *n* = 30; male Cre^+ ^= 28; female Cre^−^ *n* = 34; female Cre^+^ *n* = 16) tests. ***H***, Basal stress levels, measured as corticosterone levels at onset of dark phase of light cycle. Male Cre^−^ *n* = 11; male Cre^+ ^= 17; female Cre^−^ *n* = 12; female Cre^+^ *n* = 11. *p < 0.05, **p < 0.01, ***p < 0.001.

After developmental screening, body weights were monitored once per 8–10 d until P50 (Fig. 5*A*). A repeated-measures ANOVA shows a main effect of time (*F*_(3,228) _= 4,486.3; *p* < 0.001; *η*^2 ^= 0.98) and sex (*F*_(1,76) _= 30.08; *p* < 0.001; *η*^2 ^= 0.28) with a time*sex interaction (*F*_(3,228) _= 61.25; *p* < 0.001; *η*^2 ^= 0.45).

#### Locomotor activity

To assess whether the insertion of Cre into *Prkcd* gene affected spontaneous behaviors, we performed a battery of tests in adult Cre^+^ and wild-type Cre^−^ animals of both sexes. First, we tested whether general locomotor activity was affected by Cre insertion by screening animals in locomotor boxes and found no effect of genotype (Fig. 5*B*; *F*_(1,124)_: 1.84; *p* = 0.17) or genotype × sex interaction (*F*_(1,124)_: 2.99; *p* = 0.09). We found a main effect of time and sex, with a time × sex interaction (main effect of time bin as within-subject factor: *F*_(5,620) _= 518.11; *p* < 0.001; *η*^2^ = 0.81; main effect of sex: *F*_(1,124) _= 7.13; *p* = 0.009; *η*^2^ = 0.05; sex × time bin interaction: *F*_(5,620) _= 3.4; *p* = 0.005; *η*^2^ = 0.03). Post hoc analysis showed that female rats had higher levels of locomotion in the first time bin compared with males (*p* = 0.004). Total distance traveled (Fig. 5*C*) differed between sexes (*F*_(1,124) _= 7.53; *p* = 0.007; *η*^2^ = 0.06), without a sex × genotype interaction (*F*_(1,124) _= 2.9; *p* = 0.09).

#### Anxiety-like behavior

Considering the established role of PKCδ in anxiety-like behaviors (Botta et al., 2015; Douceau et al., 2022), we tested anxiety-like behavior using the elevated plus maze. We found no genotype effect, or genotype × sex interaction on %open time; we did find a trend for a sex difference, which was independent of genotype (Fig. 5*D*; main effect of genotype *F*_(1,124) _= 1.3, *p* = 0.25; main effect of sex *F*_(1,124) _= 3.44, *p* = 0.06; sex × genotype interaction *F*_(1,124) _= 0.017, *p* = 0.96).

#### Learning and memory

To evaluate whether insertion of Cre would affect learning and memory, we performed a novel object recognition test and found no effect of genotype or genotype × sex interaction on the discrimination index (Fig. 5*E*; main effect of genotype: *F*_(1,39) _= 0.01, *p* = 0.91; main effect of sex: *F*_(1,39) _= 0.87, *p* = 0.36; sex × genotype interaction: *F*_(1,39) _= 0.361, *p* = 0.55).

#### Pain sensitivity

Considering the established role of CeA PKCδ+ neurons in pain processing (Wilson et al., 2019; Singh et al., 2022), we performed a tail flick and a hot plate test. Neither test showed an effect of genotype or genotype × sex interaction; a sex difference independent of genotype was found in the tail flick with females showing a shorter response latency to tail flick than males, while no sex difference was found in the hot plate test (tail flick, Fig. 5*F*; main effect of genotype: *F*_(1,105)_ = 0.085, *p* = 0.77; main effect of sex: *F*_(1,105)_ = 4.86, *p* = 0.03, *η*^2^ = 0.04; genotype × sex interaction: *F*_(1,105)_ = 0.34, *p* = 0.56; hot plate, Fig. 5*G*; main effect of genotype: *F*_(1,104)_ = 0.01, *p* = 0.91, main effect of sex: *F*_(1,105)_ = 1.42, *p* = 0.23; sex × genotype interaction *F*_(1,105)_ = 0.134, *p* = 0.72).

#### Basal corticosterone levels

We tested the effect of genotype on basal corticosterone levels. Once again, we found no effect of genotype or genotype × sex difference, while finding that females showed higher corticosterone levels than males independent of genotype (Fig. 5*H*; main effect of genotype: *F*_(1,47) _= 19, *p* = 0.66; main effect of sex: *F*_(1,47) _= 65.3, *p* < 0.001, *η*^2^ = 0.58; sex × genotype interaction: *F*_(1,47) _= 0.04, *p* = 0.84).

#### Summary of baseline phenotyping

Altogether, these results suggest that, even though the insertion of the Cre-cassette affected the CeA *Prkcd* mRNA levels, this manipulation did not produce clear alterations of basal animal behavior.

### Optogenetic activation of CeL PKCδ+ neurons

The behavioral role of CeA PKCδ+ neurons has been investigated using a mouse *Prkcd-*Cre line (B. Li, 2019). To provide a functional validation of our *Prkcd-*Cre rat line, we selectively expressed ChR2-eYFP in CeA PKCδ+ neurons and optogenetically stimulated these cells during a series of tests.

#### Slice electrophysiology

**Figure 6.**
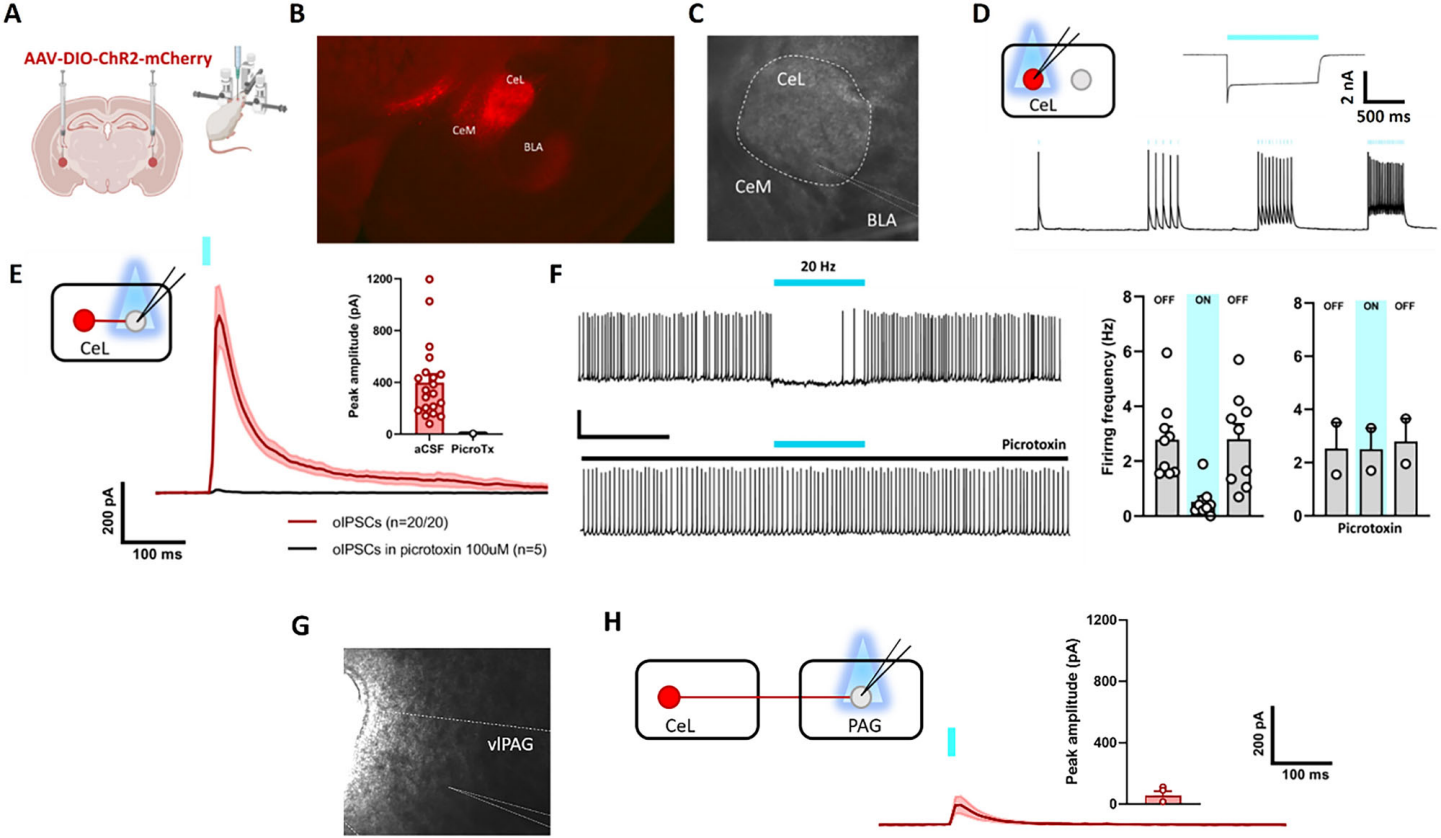
Electrophysiological properties of CeA neurons in Prkcd-cre rats. ***A***, Schematic overview of experimental setup. Prkcd-cre rats received bilateral injection of ChR2-mCherry in the centrolateral amygdala (CeL), a subdivision of the CeA. ***B***, ***C***, Representative picture showing ChR2-mCherry expression and recording locations. ***D***, Blue light stimulation evokes action potentials in ChR2-mCherry CeL neurons. ***E***, Optogenetic stimulation evokes IPSCs in recorded mCherry-negative CeL neurons, abolished by bath application of picrotoxin. ***F***, Representative traces of the firing activity recorded from mCherry-negative CeL neurons and their response to optogenetic stimulation of CeL PKCδ neurons in absence (top) or presence (bottom) of picrotoxin, and its quantification. ***G***, Representative image showing vlPAG recording locations. ***H***, Optogenetic stimulation evokes IPSCs in a subset of recorded vlPAG neurons.

First, we performed ex vivo patch-clamp electrophysiological experiments to validate the approach for optogenetic stimulation of CeA PKCδ+ neurons (Fig. 6). We found that wide-field blue light stimulation reliably evoked action potential up to 20 Hz in mCherry-positive neurons (Fig. 6*D*). Consistent with previous findings (Haubensak et al., 2010), optogenetic stimulation of CeA PKCδ+ neurons evoked postsynaptic inhibitory currents (Fig. 6*E*; 399.14 ± 65.25 pA) onto all (20/20) neighboring mCherry-negative cells and decreased their firing activity (Fig. 6*F*; *F*_(8,16) _= 4,66; *p* = 0.043). As expected, these effects were blocked by bath perfusion of picrotoxin (100 μM), confirming that they are mediated by GABA release from CeA PKCδ+ neurons (Fig. 6*E*,*F*). To further investigate the contribution of specific CeA PKCδ+ projections sites, we also evaluated the functional connectivity between these cells the vlPAG. Reminiscent of previous findings evaluating connections between these cells and the parabrachial nucleus in mice (Douglass et al., 2017), we found weak levels of connectivity, as optogenetic stimulation of CeA PKCδ+ fibers evoked IPSCs (Fig. 6*H*; 57.77 ± 25.41 pA) only in a minority of the vlPAG neurons recorded (4/13).

#### Feeding

**Figure 7.**
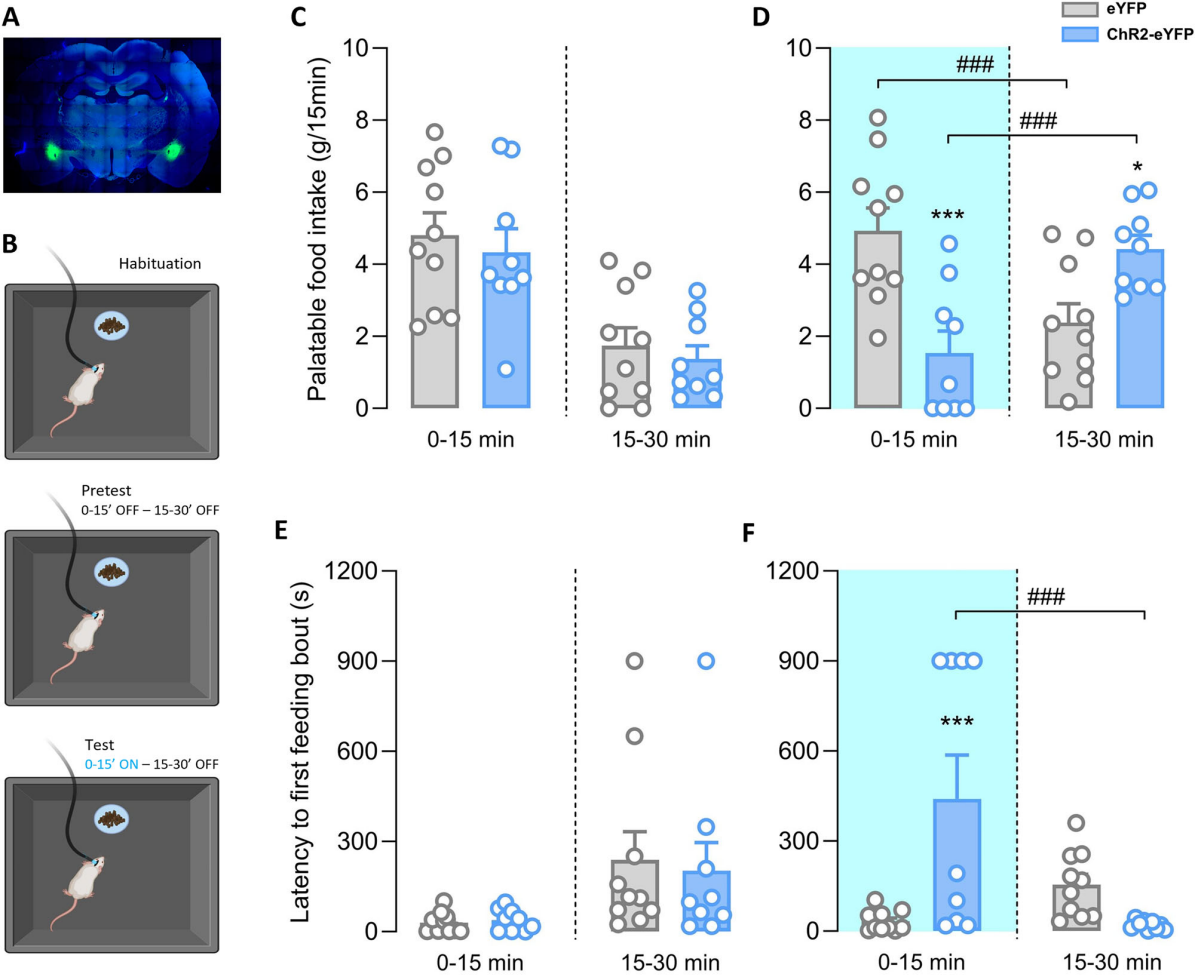
Effects of optogenetic stimulation of CeA PKCδ+ neurons on palatable food intake. ***A***, Representative images of CeA photomicrographs showing eYFP immunoreactivity (green) and DAPI (blue). ***B***, Schematic of the protocol to assess the role of CeA PKCδ+ neurons on feeding behavior. ***C–F***, Graphs showing the amount of food ingested (***C***, ***D***) and latency to the first feeding episode (***E***, ***F***) on pretest (***C***, ***E***) and test (***D***, ***F***) day by eYFP-controls (*n *= 10) and ChR2-eYFP-expressing rats (*n *= 9) at 0–15 and 15–30 min. Pretest was carried out in absence of stimulation; on test day CeA PKCδ+ neurons were stimulated only during the first 15 min.

Previous studies demonstrated that stimulation of CeA PKCδ+ neurons is anorexigenic in mice (Cai et al., 2014). We therefore evaluated the effects of optogenetically stimulating CeA PKCδ+ cells on palatable food intake in male rats. First, animals underwent 6 d of habituation to palatable food to avoid confounds due to novelty. Subsequently, they were exposed to a pretest, where food intake was measured 15 and 30 min after the beginning of the test in the absence of light stimulation (0–15 min, light OFF; 15–30 min, light OFF). Under these conditions, two-way ANOVA with group as a between-group factor (eYFP vs ChR2-eYFP) and time as a within-subject factor (0–15 vs 15–30 min) showed no main effect of group (*F*_(1,17) _= 0.40; *p* = 0.905), a main effect of the time (*F*_(1,17) _= 54.07; *p* < 0.001), and no group × time interaction (Fig. 7*C*; *F*_(1,17) _= 0.01; *p* = 0.905). Accordingly, there was no main effect of group (*F*_(1,17) _= 0.04; *p* = 0.8473), a main effect of time (*F*_(1,17) _= 8.663; *p* < 0.001), but no group × time interaction (*F*_(1,17) _= 0.12; *p* = 0.725) on the latency to the first feeding bout (Fig. 7*E*). This shows that both groups consumed palatable food mostly in the first half of the pretest and then decreased their food intake similarly in the second half of the pretest.

During the test day, conditions were maintained the same as the pretest, but CeA PKCδ+ neurons were optogenetically stimulated (10 Hz, 10 ms) during the first half of the test (0–15 min, light ON; 15–30 min, light OFF). There was no main effect of group (*F*_(1,17) _= 1.06; *p* = 0.318) or time (*F*_(1,17) _= 0.15; *p* = 0.704), but a significant group × time interaction (*F*_(1,17) _= 41.59; *p* < 0.001) on palatable food intake (Fig. 7*D*). Similarly, two-way ANOVA showed no main effect of group (*F*_(1,17) _= 3.57; *p* = 0.076) or time (*F*_(1,17) _= 4.37; *p* = 0.052), but a significant group × time interaction (*F*_(1,17) _= 14.41; *p* = 0.0014) on the latency to the first feeding bout (Fig. 7*F*). Post hoc analysis showed that ChR2-eYFP rats consumed significantly less palatable food and increased the latency for the first feeding bout during optogenetic stimulation (0–15 min) compared with control animals (ChR2-eYFP at 0–15 min vs eYFP at 0–15 min: food intake *p* < 0.001; latency *p* = 0.002). Upon cessation of optogenetic stimulation, ChR2-eYFP rats also showed a rebound of feeding behavior, eating significantly more food and decreasing the latency to the first feeding bout in comparison with the first half of the test (ChR2 eYFP at 15–30 min vs ChR2-eYFP at 0–15 min: food intake *p* = 0.001; latency *p* = 0.004).

#### Real-time place aversion

**Figure 8.**
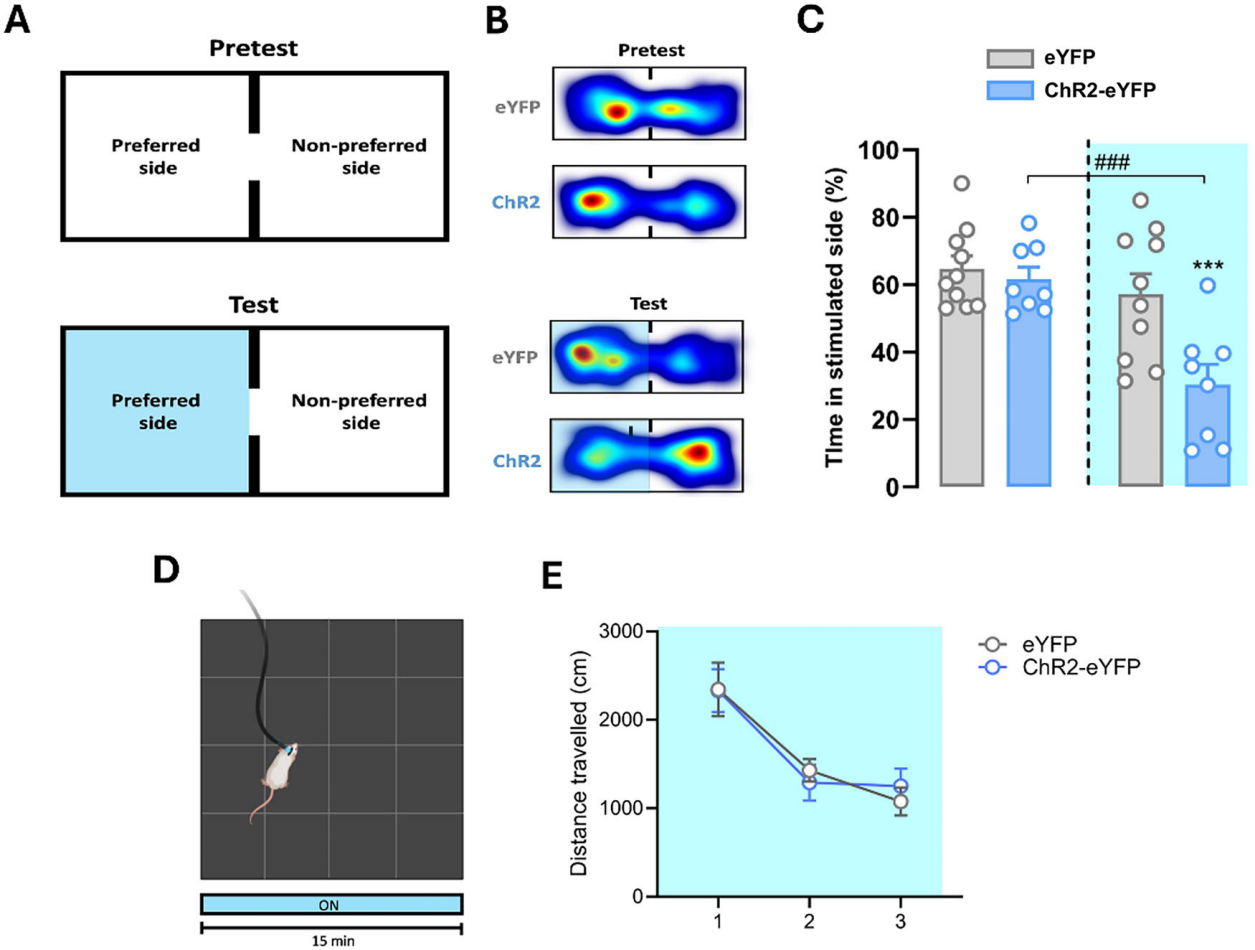
Effects of optogenetic stimulation of CeA PKCδ+ neurons on RTPA and locomotor activity. ***A***, Schematic of the protocol to assess RTPA induced by optogenetic stimulation of CeA PKCδ+ neurons. ***B***, Representative heat maps, showing place preference during pretest and test in eYFP-controls (*n *= 10) and ChR2-eYFP-expressing rats (*n *= 8). ***C***, Histogram showing the percentage time spent in the stimulated side during pretest and test by eYFP-controls and ChR2-eYFP-expressing rats. ***D***, Schematic of the protocol to assess the effects of CeA PKCδ optogenetic stimulation on the open field test. ***E***, Graph showing the effect of optogenetic stimulation on locomotor activity on eYFP-controls and ChR2-eYFP-expressing rats.

CeA PKCδ+ neurons are also known to be activated by a wide repertoire of aversive stimuli and to induce place aversion when artificially stimulated (Cui et al., 2017; K. Yu et al., 2017). To confirm that optogenetic stimulation of CeA PKCδ+ neuron promotes aversion, we tested male rats in a biased real-time place preference task. Optogenetic stimulation was paired with the chamber each single animal preferred during a pretest carried out without light stimulation (Fig. 8*A*). Two-way ANOVA with group as a between-group factor (eYFP vs ChR2-eYFP) and time as a within-subject factor (Pretest vs Test) showed a main effect of group (*F*_(1,16) _= 6.21; *p* = 0.024), a main effect of the time (*F*_(1,16) _= 23.55; *p* < 0.001), and a group × time interaction (Fig. 8*C*; *F*_(1,16) _= 8.7896; *p* = 0.009). Post hoc tests showed that during test, ChR2-eYFP-expressing rats spent significantly less time in the light-paired chamber compared with eYFP control rats (*p* = 0.005) and to their pretest baseline (*p* < 0.001).

To confirm that the observed effects were not a mere consequence of changes in locomotor activity, we tested the effect of CeA PKCδ+ neurons optogenetic stimulation during an open field test. By using a repeated-measures ANOVA, there was no main effect of group (*F*_(1,15) _= 0.001; *p* = 0.97), a main effect of the time (*F*_(2,30) _= 31.88; *p* < 0.001), but no treatment × time interaction (Fig. 8*E*; *F*_(2,30) _= 0.509; *p* = 0.609). This shows that optogenetic stimulation of CeA PKCδ+ neurons did not affect locomotor behavior.

#### Conclusion of functional validation tests

Collectively, these findings recapitulate previous results obtained using a *Prkcd-*Cre mouse line suggesting that this novel *Prkcd-*Cre rat line might represent a valuable complementary tool to investigate the role of PKCδ+ neurons in behaviors in which the use of a rat model is preferable.

## Discussion

We describe a novel PKCδ-Cre rat line and provide data supporting its utility for functional studies. Mouse models have been key to advancing the understanding of the role PKCδ+ neurons play in CeA microcircuitry (Janak and Tye, 2015), but there are anatomical, physiological, and behavioral differences between mice and rats of relevance for research on neuropsychiatric conditions (Parker et al., 2014; Ellenbroek and Youn, 2016). For instance, intravenous self-administration, a foundational technique of addiction research, is largely limited to short-term studies in mice due to limited duration of catheter patency but can be carried out over months in rats, allowing studies of long-term processes such as extended access-induced escalation (Ahmed and Koob, 1998), emergence of individual vulnerability (Deroche-Gamonet et al., 2004), or relapse and incubation of craving (Grimm et al., 2001; Vengeliene et al., 2014; Venniro et al., 2020). Furthermore, over the 15–20 million years since the common ancestor of rats and mice, many complex behaviors, including those that are impulsivity related (Parker et al., 2014) and social (Vestal, 1977; Kummer et al., 2014; Golden et al., 2017; Venniro et al., 2018), have diverged between these species. Thus, our rat model importantly expands the range of experimental strategies to examine the mechanistic role of PKCδ+ neurons in behavior and physiology. It also enables the consistency of these mechanisms to be examined across species, with the potential to improve translatability to humans.

### Cre-insertion into the *Prkcd* locus results in selective Cre expression in CeA PKCδ+ neurons

We found the expected PKCδ expression in the CeL of *Prkcd-*Cre animals, as described in rats (Amano et al., 2012) and mice (McCullough et al., 2018), and found that Cre was selectively expressed in PKCδ+ CeA neurons. Insertion of the Cre-cassette into the *Prkcd* locus resulted in a modest increase of *Prkcd* expression, particularly in the CeA of male, but not female Cre^+^ rats. Cas9 can create double-strand breaks in proximity of a targeted gene (Shin et al., 2017), resulting in activation of the endogenous repair systems, and potentially creating unexpected short deletions, insertions, and point mutations (Shin et al., 2017; Yang et al., 2023). Similarly increased expression of the targeted gene was recently reported in another CRISPR/Cas9 knock-in rat line that expresses Cre under the control of the μ-opioid receptor promoter (Bossert et al., 2023).

The reasons for a primarily male-specific increase of PKCδ expression in our model are unclear. Studies in non-neuronal cells have demonstrated that testosterone promotes PKCδ expression, while more complex, reciprocal interactions with PKCδ have been shown for estrogen, dependent on the subtype of estrogen receptor predominantly expressed by the cell. Estrogen receptor alpha suppresses PKCδ expression, while the opposite effect is mediated by the beta subtype. PKCδ then feeds back to modulate the transcriptional activity of estrogen (Cutler et al., 1994; Lahooti et al., 1998; Shanmugam et al., 1999; O'Mahony et al., 2009). Only limited data are available from neuronal cells. In primary cultures of rat cerebrocortical neurons, estrogen was shown to increase global protein kinase C activity, but these experiments did not specifically examine effects on the PKCδ isoform (Cordey et al., 2003).

Thus, for now, molecular mechanism mediating the modest, primarily male-driven increase in PKCδ expression in our model remains unknown. Neither global nor sex-dependent consequences of the transgene insertion were found in our phenotype screen. Nevertheless, the expression data suggest that caution is needed to minimize potential confounds from effects on PKCδ expression and from potential sex-specific expression differences. Specifically, experiments using the novel rat line should use Cre+ rats both as experimental animals and controls, and any effects observed should be examined for possible sex dependence.

### Anterograde tracing of CeA PKCδ+ neuron projections replicates findings in mouse

Anterograde tracing of CeL PKCδ+ neuron projections confirmed output targets previously reported in mice (Penzo et al., 2015; Schiff et al., 2018; Fu et al., 2020). Specifically, we identified local inputs to the CeM, as well as monosynaptic projections to the BNST, and the vlPAG. We functionally confirmed the latter projection using ChR2-assisted circuit mapping and found modest connectivity to the vlPAG. This may depend on CeA PKCδ+ neurons that selectively target specific subtype of vlPAG neurons. However, CeA PKCδ+ neurons also synapse onto PAG-projecting CeM neurons and control their activity (Haubensak et al., 2010), and it is therefore likely that modulation of vlPAG neuronal function from CeA PKCδ+ is primarily mediated by a multisynaptic mechanism. CeL PKCδ+ neurons showed robust projections to the substantia nigra (SN) and the ventral tegmental area (VTA), in line with previous data obtained in mice (K. Yu et al., 2017; Fu et al., 2020). We also observed a strong projection to the mesopontine tegmentum, especially the pedunculopontine tegmentum (PPTg) and the microcellular tegmentum. It is well established that the mesopontine tegmentum is functionally linked to the dopaminergic system. Studies show that cholinergic neurons of the PPTg alter reinforcement and motor activity through parallel pathways to the VTA and SN, respectively (Mena-Segovia et al., 2008; Jenkinson et al., 2009; Benarroch, 2013). In line with this, increased activity of cholinergic PPTg neurons in rats is related to drug seeking and a positive emotional valence to previous aversive situations such as increased time spent in aversive places (Xiao et al., 2016).

### Cre insertion into the *Prkcd* locus does not alter spontaneous behaviors or corticosterone levels

We performed developmental screening in Cre^+^ and Cre^−^ littermates from P4 to P21 (see Extended Data) and an extensive behavioral characterization in adult Cre^+^ and Cre^−^ animals of both sexes to assess potential effects of the Cre insertion on behaviors in which CeA PKCδ+ neurons are involved. We found no effects of genotype on anxiety-like behaviors, memory function, or pain responses. We also did not find any effect of genotype on general locomotion or corticosterone levels. In all these assays, effects of genotype were absent both in overall and in sex-specific analyses. Together, our behavioral characterization indicates that insertion of the Cre cassette did not alter spontaneous activity levels or learning, nor did it influence behaviors in which a role of CeA PKCδ has been established, such as anxiety-like or pain-related behaviors. Because CeA PKCδ mRNA levels were increased by Cre insertion, we cannot exclude that some aspects of physiology or behavior that we did not examine might be affected by our Cre insertion. This includes functions other than those controlled by CeA, as PKCδ is widely expressed outside this structure, both within the brain and peripheral organs (Goldberg and Steinberg, 1996; Haubensak et al., 2010). This makes it important that Cre^+^ littermates are used as controls in studies using this line to manipulate the activity of PKCδ+ neurons.

### Optogenetic activation of CeA PKCδ+ neurons replicates prior findings in mouse

We found that optogenetic stimulation of CeA PKCδ+ neurons significantly reduced palatable food intake in rats, recapitulating and extending previous mouse findings. Using mice, it has been shown that CeA PKCδ+ neurons are activated by anorexigenic signals and that optogenetic stimulation of their activity results in decreased intake of regular chow (Cai et al., 2014). This effect was readily reversible, as animals exhibited a rebound of feeding right after optogenetic stimulation ceased. Our results replicate these findings and extend them by showing that CeA PKCδ+ neuron activity also reduces consumption of palatable food in nondeprived animals. In addition, we found that activation of CeA PKCδ+ neurons induced RTPA, confirming a consistent body of literature showing that these neurons encode aversive signals in mice (Cui et al., 2017; K. Yu et al., 2017).

We have focused on the lateral subdivision of the central amygdala, but PKCδ+ neurons are widely expressed throughout the brain (Goldberg and Steinberg, 1996; Haubensak et al., 2010), and our model will offer opportunities for functional studies involving those structures. Within CeA, a functional role of PKCδ+ neurons was initially described in fear conditioning, anxiety-related behaviors, and appetite regulation (Ciocchi et al., 2010; Haubensak et al., 2010; Douglass et al., 2017), while more recent work by our group and others has implied this neuronal population in addiction-related behaviors (Venniro et al., 2018; Venniro and Shaham, 2020; Domi et al., 2021; Dilly et al., 2022). Providing data that expand the mechanistic understanding of the role that CeA PKCδ+ neurons play in these behaviors is beyond the scope of the present paper. However, we provide a detailed characterization and validation of the line that will enable this type of studies.

### Conclusion

Our findings consistently support the utility of the new *Prkcd-*Cre rat line for functional studies. The model is of particular interest for studies of addiction-related behaviors, since CeA PKCδ+ neurons play a role in compulsive alcohol- and opioid-related behaviors ( Chaudun et al., 2024). PKCδ expression in the CeA is evolutionarily conserved, including humans (B. Yu et al., 2023), suggesting a translational utility of rodent studies. The *Prkcd-*Cre line offers a tool to examine the functional role of PKCδ+ neurons and related circuitry in behaviors where rat models are of value.
